# Microenvironmental changes co-occur with mosaic somatic clonal expansions in normal skin and esophagus tissues

**DOI:** 10.3389/fonc.2022.1021940

**Published:** 2022-12-01

**Authors:** C. Munugula, J. Hu, E. Christodoulou, V. Yellapantula

**Affiliations:** ^1^ Center for Personalized Medicine, Children’s Hospital Los Angeles, Los Angeles, CA, United States; ^2^ Keck School of Medicine, University of Southern California, Los Angeles, CA, United States

**Keywords:** ageing and cancer, microenvironment, genomics, computation biology, bioinforamtics

## Abstract

The presence of somatic mutations, previously identified in cancers, are being increasingly recognized in normal tissues. While the role of microenvironment (ME) in tumor progression is well understood, the changes that occur in the microenvironment of normal tissues that harbor somatic mutations has not been systematically studied. Here, using normal RNA-Seq data accrued from 6544 samples across 27 tissue types from Genotype-Tissue Expression (GTEx) project, we studied the association of microenvironmental changes in the presence of somatic clonal expansions of previously implicated cancer genes. We focused our analysis on skin and esophagus since they have the highest number of samples and mutation burden together. We observed changes in microenvironmental cell-types previously implicated in tumor progression including endothelial cells, epithelial cells, pericytes, fibroblasts, chondrocytes, among others. The Epithelial-Mesenchymal-Transition (EMT) pathway is dysregulated in both skin and esophagus, along with increased hypoxia scores in samples with clonal expansions. These results suggest that microenvironmental changes play an important role in clonal expansions and potentially the initiating stages of cancer progression. Studying these changes may provide new avenues for early intervention of cancer, for targeted therapies, or enhance activities of conventional therapies.

## Introduction

Cancers arise due to the sequential accrual of mutations that confer a selective advantage to the cells harboring them. Clonal hematopoiesis mutations, ie. mutations that cause clonally expanded hematopoietic stem cells in normal individuals, present an increased risk for developing myeloid malignancies. However, additional changes are necessary for cancer progression (1, 2). The recent discovery of clonal expansions of mutant cells in skin and esophagus suggests the pervasiveness of this process across tissue types (3, 4).

Evading immune destruction, epigenetic reprogramming of the microenvironment, and angiogenesis (5), are hallmarks of cancer, all of which rely on the microenvironment (ME). For example, to provide nutrients and oxygen to the proliferating cells, new endothelial cells are assembled into tubes; additionally sprouting of new vessels from existing blood vessels occurs (6). This process is akin to processes in embryogenesis, however, unlike in embryogenesis, this “angiogenic-switch” is always left “on” during tumorigenesis. Tumor-associated inflammatory responses, which were earlier thought to work to eradicate tumors, also function as tumor-promoting by providing bioactive molecules conducive to a proliferative microenvironment milieu (7). Inflammation anecdotally is observed in the earliest stages of cancer progression. The Pre-Cancer Atlas (PCA) underscores the importance of understanding the ME changes in pre-malignant samples as an important step for effective risk stratification and intervention strategies (8). Others have also observed pro-inflammatory signaling in adjacent normal tissues of tumors (9). Yet, the changes in the ME that occur during clonal expansions have not been systematically studied.

Bioinformatics methods also make it possible to identify somatic mutations from RNA-Seq data (10–12). The application of this approach to normal sequencing data from GTEx showed a pervasive presence of clonal expansions in normal tissues. Of note, hotspot, mutations in *TP53, PIK3CA*, *KRAS, GNAS*, among others, were previously identified in normal tissues (10). The dN/dS ratios of the identified somatic mutations were indicative of a selective advantage of the clones harboring them, suggesting an ideal resource to observe the earliest changes in the ME that occur with such clonal expansions. Recent developments in computational methods, allow the deconvolution of microenvironment composition, including immune and stromal cells, from bulk RNA-seq data (13, 14). Here, using GTEx RNA-Seq data from 27 normal tissue types and 6544 samples, we identified esophagus and skin to have the highest mutation rate and number of samples. We report changes in the microenvironment that occur in these tissues that have somatic mutation-related clonal expansions.

## Methods

### Microenvironment content enumeration and classification

Previously reported methods have performed an extensive analysis of commonly used microenvironment deconvolution methods: 1) XCell 2) CIBERSORTx (15, 16). Despite the differences in the deconvolution approaches, earlier studies show a high correlation of the immune and stromal scores (15). GTEx Version v8, normalized TPM values were downloaded from GTEx. Samples were limited to those which had somatic mutations previously called by Yizhak et al (10). Deconvolution was performed using CIBERSORTx-Relative, CIBERSORTx-Absolute and XCell. CIBERSORTx significance scores were generated using 1000 permutations and quantile normalization disabled. For XCell, as previously suggested (15), samples were separated by tissue, and deconvolution was performed using the XCell R script using the default settings.

### Classifying samples by mutations status

**Figure 1:**
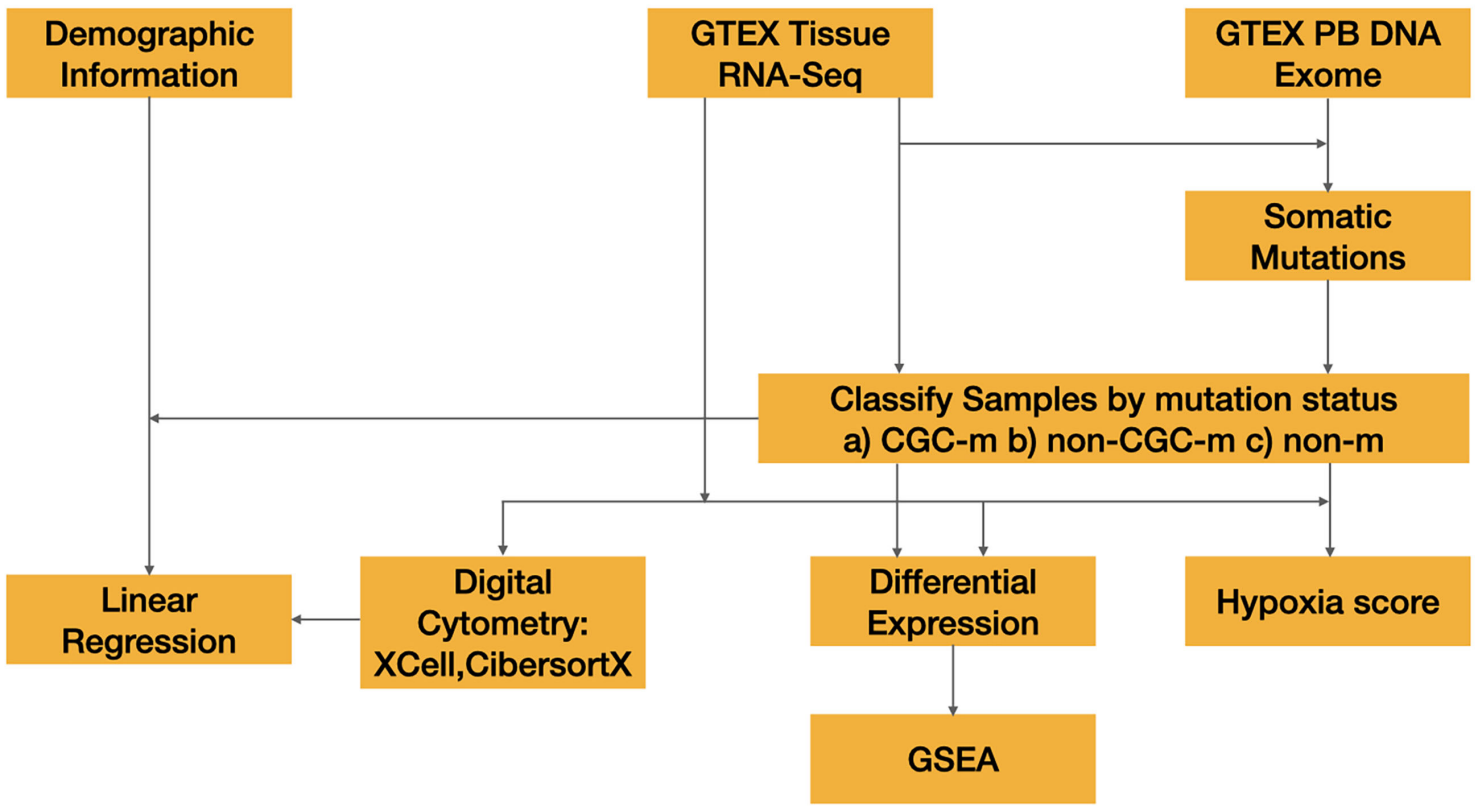
Overview of methods workflow used to detect microenvironment changes in normal tissues.

Mutations previously called by Yizhak et al. were downloaded through dpGAP (Study Accession: phs000424.v8.p2) (10). Mutations were limited to non-synonymous mutations namely, missense, nonsense, nonstop, splice site and start codon mutations. Subsequently, cancer gene census (CGC) genes defined by COSMIC v94 were downloaded. Each sample was then classified into one of the following three categories 1) non-synonymous mutation in a cancer gene present (CGC-m) 2) non-synonymous mutation in a non-cancer gene present (non-CGC) 3) no non-synonymous mutation present (non-m) (**Figure 1**). Additionally, we classified samples by their presence of a non-synonymous mutation in Significantly Mutated Genes (SMG-m) previously identified in esophageal and cutaneous melanoma (17, 18).

### Linear model

A linear regression was fit for each cell type and tissue type by regressing cell type abundance scores from XCell or CIBERSORTx on mutation status (CGC-m or non-m), along with age and sex as covariates. T-tests of estimated coefficients were performed, and p-values were adjusted to control for false discovery rate using the Benjamini-Hochberg correction. The model was fit in R using the lm function as shown below and p-values were adjusted using the p.adjust() function.


model<− lm(Cell Type Abundance Score~Group+Sex+Age)

### DEG and pathway analysis

Raw gene counts from GTEx v8 were downloaded. Within a given tissue type, differential gene expression testing was performed using Deseq2 between CGC-m and non-m samples as defined above. Genes with p-adjusted value > 0.01, base-mean value< 200 and absolute log2 fold-change< 0.2 were excluded for downstream analysis. Orthogonally, a Wilcoxon rank-sum test was run between the CGC-m and non-m groups. Genes significantly differentially expressed (DE) by both approaches was used for downstream analysis.

## Results

### Changes in microenvironment with somatic mutations status

**Figure 2:**
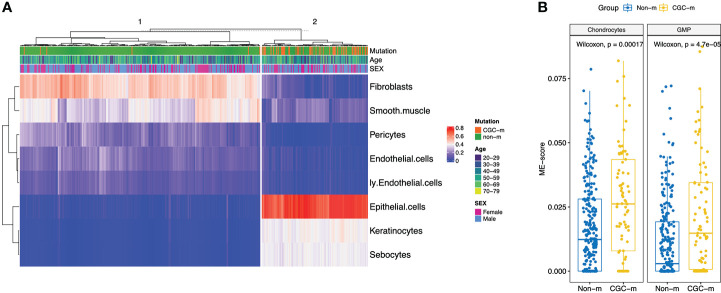
XCell scores of significantly different microenvironment cell-types, between CGC-m and non-m groups, identified using linear regression with age and gender as covariates. **(A)** Heatmap of significantly different ME scores in Esophagus previously implicated in cancer progression **(B)** Boxplots of significantly different ME scores in skin.

We looked at the effect of how expansion of somatic mutations are associated with changes in the microenvironment. For this analysis, we limited the studied tissue types to skin and esophagus due to presence of the highest non-synonymous mutation burden in cancer genes and the highest number of samples present (Esophagus: N=600, CGC-m rate = 0.13; Skin: N=512, CGC-m rate = 0.3; **Supplementary Table S1**). Earlier reports have shown that age and especially sex are significantly associated with differences in microenvironment (ME) (15). For example, there is an increased lymphocyte content in breast and thyroid tissues in females when compared to males. Interestingly, women portend a higher prevalence of auto-immune and neoplastic disorders suggesting that such ME differences could play a role in pathology (15). Several age-associated differences such as an increase in lymphocytes and myeloid cells in the artery were associated with age (15). Secondly, it is well known that somatic mutations in healthy individuals is associated with age (15). To account for such associations, we use a linear regression model using age and sex as covariates. Of relevance, for esophagus, we observed the most significant association (p-adjusted< 0.01) of CGC-m status with an increased presence of epithelial cells, keratinocytes, sebocytes and reduced expression of endothelial and lymphatic endothelial cells, pericytes, smooth muscles and fibroblasts (**Figure 2A**, **Supplementary Table S3**). Unsupervised hierarchical clustering using significant ME cell-types classified 50/56 (89%) CGC-m cases into cluster same cluster. Interestingly, recent studies in Barret’s Esosphagus (BE), which is a precursor to esophageal cancer, show that it is characterized by the occurrence of specialized columnar epithelium (19). Lymphatic endothelial cells and their cross-talk with pericytes were shown to be important for microvasculature for mature and were both reduced in CGC-m samples (20). For skin, we observed a significant association of CGC-m with increased Chondrocytes and Granulocyte-Macrophage Progenitor (GMP) cells (p-adjusted< 0.1) (**Figure 2B**, **Supplementary Table S4**). Chondrocytes respond to outside stimuli and tissue damage by proliferating and secreting extracellular matrix that maintains and sustains cartilage (21).GMP cells which are precursors to monoblasts and myeloblasts are also elevated in CGC-m samples.

### Differential gene expression and pathway analysis

**Figure 3:**
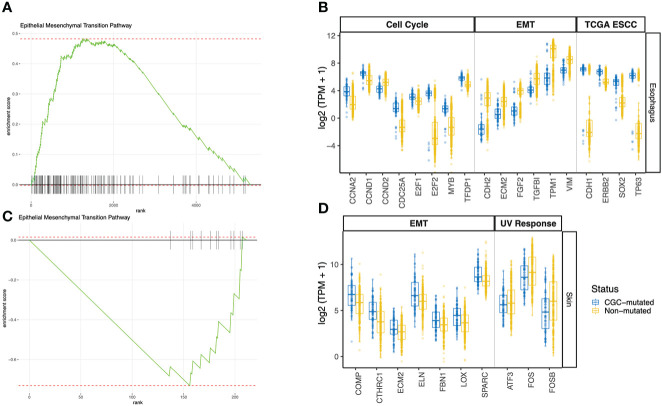
GSEA Pathway analysis using differentially expressed genes between CGC-m and non-mutated groups. **(A, C)** Shows GSEA enrichment of EMT pathway in esophagus and skin **(B)** Boxplots of gene expression (log2 TPM +1) between CGC-m and non-m cases for significantly differentially expressed genes (p-adjusted<= 0.01) genes implicated in the EMT pathway, cell cycle pathway and esophageal carcinomas previously reported by TCGA. **(D)** Boxplots of gene expression (log2 TPM +1) between CGC-m and non-m cases for significantly differentially expressed gens (p-adjusted<= 0.01) genes implicated in the EMT and UV response pathways.

We looked at genes that are differentially expressed between the CGC-m and non-m cases in skin and esophagus. Interestingly, differential gene expression testing between CGC-mutant and non-mutant esophagus samples identified genes commonly aberrant in carcinomas. *ERBB2, CDH1, SOX2*, and *TP63* were all significantly overexpressed (adjusted p-value< 0.01) in the CGC-mutant samples (**Figure 3B**). No mutations were identified in these genes suggesting that alternate mechanisms could potentially drive clonal expansions in normal tissues. This is consistent with previous reports that showed copy-number aberrations in normal tissues (4).

Pathway analysis of DEGs revealed a dysregulated Epithelial-Mesenchymal-Transition (EMT) pathway in both Skin and Esophagus tissues (**Figures 3A–D**). The EMT process is a highly dynamic process that enables interconversion of epithelial cells to mesenchymal-like states which have stem-cell like properties and a variety of intermediate states. In neoplasia, mesenchymal cells are capable of increased resistance to destruction by the adaptive immune system. An activated EMT pathway also results in degradation of cell-cell adhesive junctions and reorganization of extra-cellular matrix (22). Interestingly, in the esophagus, ECM2 which encodes extra-cellular matrix protein and is implicated in involvement with cell-ECM recognition is downregulated in samples with CGC-m compared to the non-m cohort. EMT transcription factors *ZEB1* represses *CDH1* thereby orchestrating EMT programs (23). *ZEB1* however is under expressed while *CDH1* is over-expressed in CGC-m samples in the esophagus. Other genes previously implicated in the EMT pathway and downregulated in CGC-m esophagus include *CDH2, FGF2, TGFBI, TPM1*, and *VIM* (**Figure 3B**) (24). These patterns of expression along with increased epithelial cells in CGC-m are suggestive of a repressed EMT program or perhaps a Mesenchymal Epithelial Transition (MET) phenotype. Of relevance, the cell-cycle/G2M pathway in the esophagus is also dysregulated (**Supplementary Figure 1**). Cyclin D1 which is essential for a cell to enter the G1 phase, transcription factors *E2F1* and *E2F2* required for a cell to progress to the S phase are all overexpressed in CGC-m samples (25). Other dysregulated genes in the cell cycle pathway include *CCNA2, CCND2*, and *MYB* among others (**Figure 3B**). Between the SMG-m and non-m groups, as seen in CGC-m, we also observed dysregulation of EMT and G2M pathway (**Supplementary Figure 2B, C**).

*ECM2*, whose function is known to promote matrix assembly and cell adhesiveness is dysregulated in the esophagus as previously described but also in the skin (26). However, interestingly, unlike in the esophagus, *ECM2* in the skin is upregulated in CGC-m cases. This is consistent with other EMT pathway genes in the skin (24); *COMP, CTHRC1, ECM2, FBN1, LOX*, and *SPARC* were all upregulated in samples with a mutation in a cancer gene. In the skin, the UV response pathway is also altered (**Supplementary Figure 1**). c-fos whose activation is the hallmark of response to UV is incidentally downregulated in CGC-m cases. Other key genes implicated in UV response *ATF3*, and *FOSB* among others are also downregulated in mutant cases (**Figure 3D**). In skin, only 7 samples were present in the SMG-m category which did not yield any significant results due to the small number of samples in this group.

### Hypoxia associated with clonal expansions

**Figure 4:**
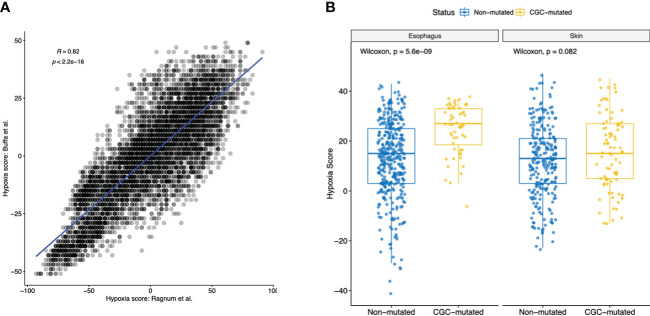
Hypoxia scores in all normal tissues using two commonly used gene signatures. **(A)** Shows a high Pearson’s correlation of 0.82 between the Hypoxia scores generated using gene signatures described in Ragnum et al. gene and Buffa et al. **(B)** Boxplot showing significant difference (Wilcoxon p-value< 0.1) in hypoxia scores of normal tissues between the CGC-m and non-mutated groups.

Hypoxia has been shown to play an important role in cancer progression, therapy resistance and metastatic growth. Hypoxia-rich environments were implicated in causing “angiogenic-switch” which dysregulated neovascularization that promotes tumor growth (27). Hypoxic environments were also shown to promote cell mobility and metastatic abilities through acquisition of epithelial-to-mesenchymal phenotype (27). Given that we identified dysregulated EMT-pathways in both Esophagus and Skin, along with the dysregulation of endothelial cells and pericyte in the ME, we hypothesized that hypoxia could play an important role in early clonal expansions. Using hypoxia gene signatures, we generated hypoxia scores for all samples using Ragnum et al. and Buffa et al. hypoxia gene signatures previously described (28). We observed a high correlation of Hypoxia scores identified using both signatures (p-value< 0.01, Pearson’s corr. = 0.82, **Figure 4A**, **Supplementary Table S2**). Next, we observed differences between the CGC-m group and the non-m group and secondly the SMG-m and the non-m groups. Interestingly, we observed significant enrichment of hypoxia scores in both esophagus and skin (Wilcoxon p-value: esophagus<0.01 and<0.01, skin=0.08, 0.06 for CGC-m and SMG-m respectively) suggesting early initiating roles of hypoxic environments in clonal expansions (**Figure 4B**, **Supplementary Figure 2A**).

## Discussion

There is a growing body of research investigating genetic aberrations in normal tissues, dysplasia and pre-cancerous lesions with the goal of charting the process of ageing and cancer progression (8). Recent studies in various tissues including skin, colon, esophagus among others predominantly investigated the DNA of the aberrant cells (3, 4, 29). While somatic mutations are age and tissue type-dependent, in skin and esophagus, approximately 30-50% of the cells in middle-aged individuals harbor driver mutations without presentation of cancer (3). Initiatives such as the PreCancer Atlas (PCA) acknowledge the importance of studying the microenvironment in understanding how precancerous lesions evolve into cancers (8).

Herein using RNA-Seq samples from GTEx consortium, we investigated the microenvironmental differences in samples that harbor a non-synonymous mutation in a cancer gene with those without non-synonymous mutations.

Focusing on skin and esophagus tissues, we identified changes in key microenvironment components in both tissues. Clustering samples by the microenvironment score differences classified 89% (50/56) of the CGC-m samples in the same cluster validating our hypothesis that key microenvironment changes occur with clonal expansions. Studying such changes through progress will be critical for our improvement of understanding of cancers. The EMT pathway was dysregulated in both skin and esophagus and its dysregulation could be an essential mechanism for cancer progression. Taken together, these findings, along with elevated hypoxia scores in CGC-mutated samples, suggest that angiogenic-switch could be a mechanism occurring early during cancer progression.

The study has the following limitations. Firstly, previous studies which sequenced normal DNA samples identified somatic copy number changes along with mutations (3, 4, 29). In this study, we were able to classify samples only through their mutation status and it’s a technical limitation of the study to not be able to robustly call such changes from RNA-Seq. Secondly, current deconvolution approaches rely on signatures of differentiated cells. However, patterns of de-differentiation are a common mechanism of cancer progression and metastasis. Hence, the approaches used in this study are not able to deconvolute dedifferentiated cell states which might have important implications for cancer progression. Single-cell sequencing of normal cells would aid such discovery. Lastly, the study fails to pinpoint the microenvironment states and mutations that lead to overt presentation of cancer. Studying such changes requires follow up biopsies and various stages of disease progression which are hard to obtain.

Despite these challenges, these results suggest that microenvironmental changes play an important role in the initiating stages of cancer progression and are tissue specific. Studying these changes may provide new avenues for early intervention of cancer, for targeted therapies, or enhance activities of conventional therapies.

## Data availability statement

Publicly available datasets were analyzed in this study. This data can be found here: https://gtexportal.org/home/.

## Ethics statement

The studies involving human participants were reviewed and approved by GTEX Consortium. Written informed consent to participate in this study was provided by the participants’ legal guardian/next of kin. The animal study was reviewed and approved by GTEX Consortium.

## Author contributions

VY designed the study, collected, and analyzed the data. CM and JH collected data and analyzed the data. CM, EC, and VY wrote the paper. All authors contributed to the article and approved the submitted version.

## Funding

We thank Childrens Hospital Los Angeles, Pathology and Lab Medicine Fund (8231000-TUA010247) for supporting Chetan Munugula.

## Conflict of interest

The authors declare that the research was conducted in the absence of any commercial or financial relationships that could be construed as a potential conflict of interest.

## Publisher’s note

All claims expressed in this article are solely those of the authors and do not necessarily represent those of their affiliated organizations, or those of the publisher, the editors and the reviewers. Any product that may be evaluated in this article, or claim that may be made by its manufacturer, is not guaranteed or endorsed by the publisher.
