# Supplementary figures and images for: Microenvironmental changes co-occur with mosaic somatic clonal expansions in normal skin and esophagus tissues
Source: Front Oncol. 2022 Dec 1;12:1021940. doi: 10.3389/fonc.2022.1021940 (PMC9751946; doi:10.3389/fonc.2022.1021940)

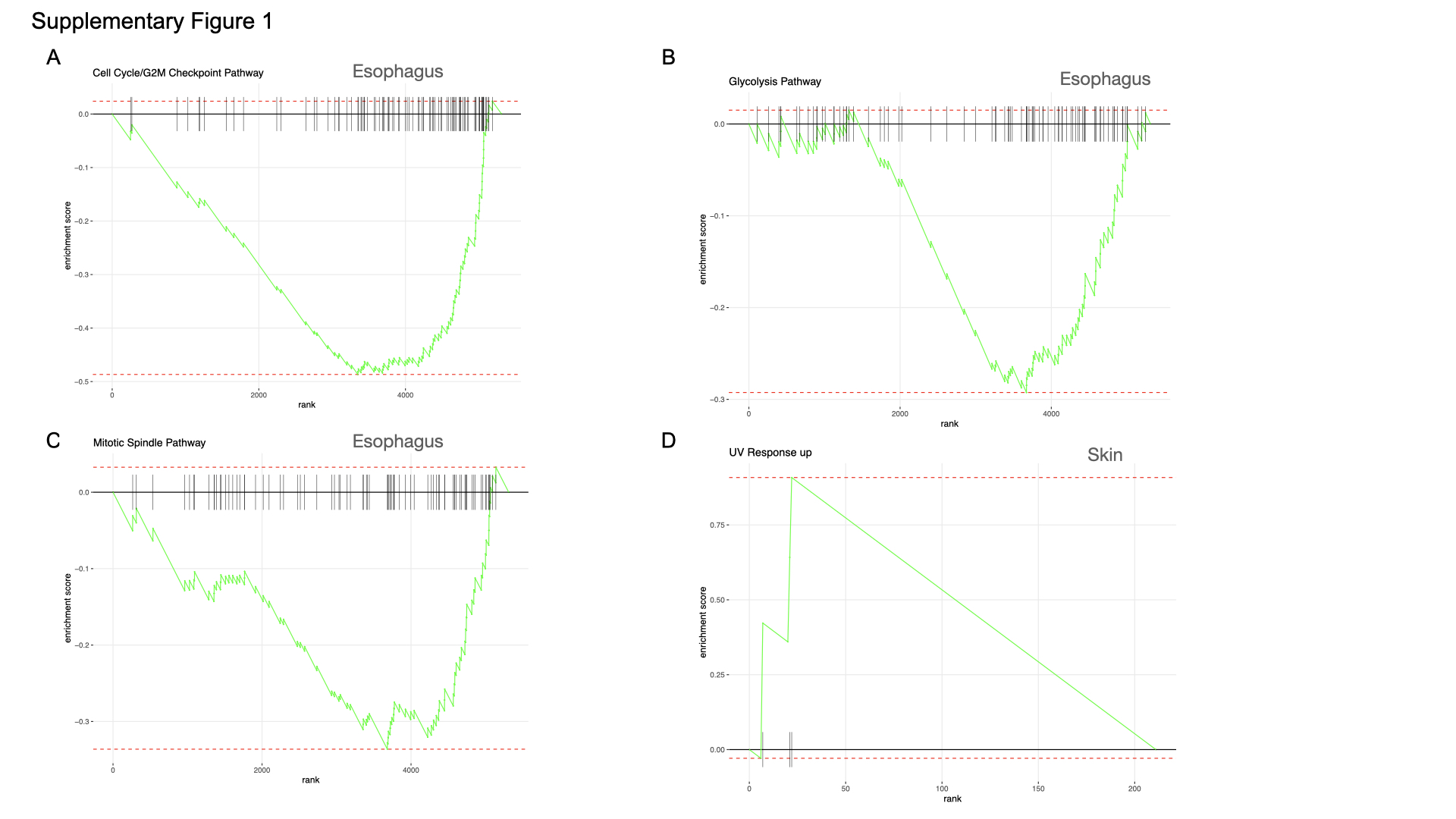

Supplement: Supplementary file 2 [file Image_1.jpeg]

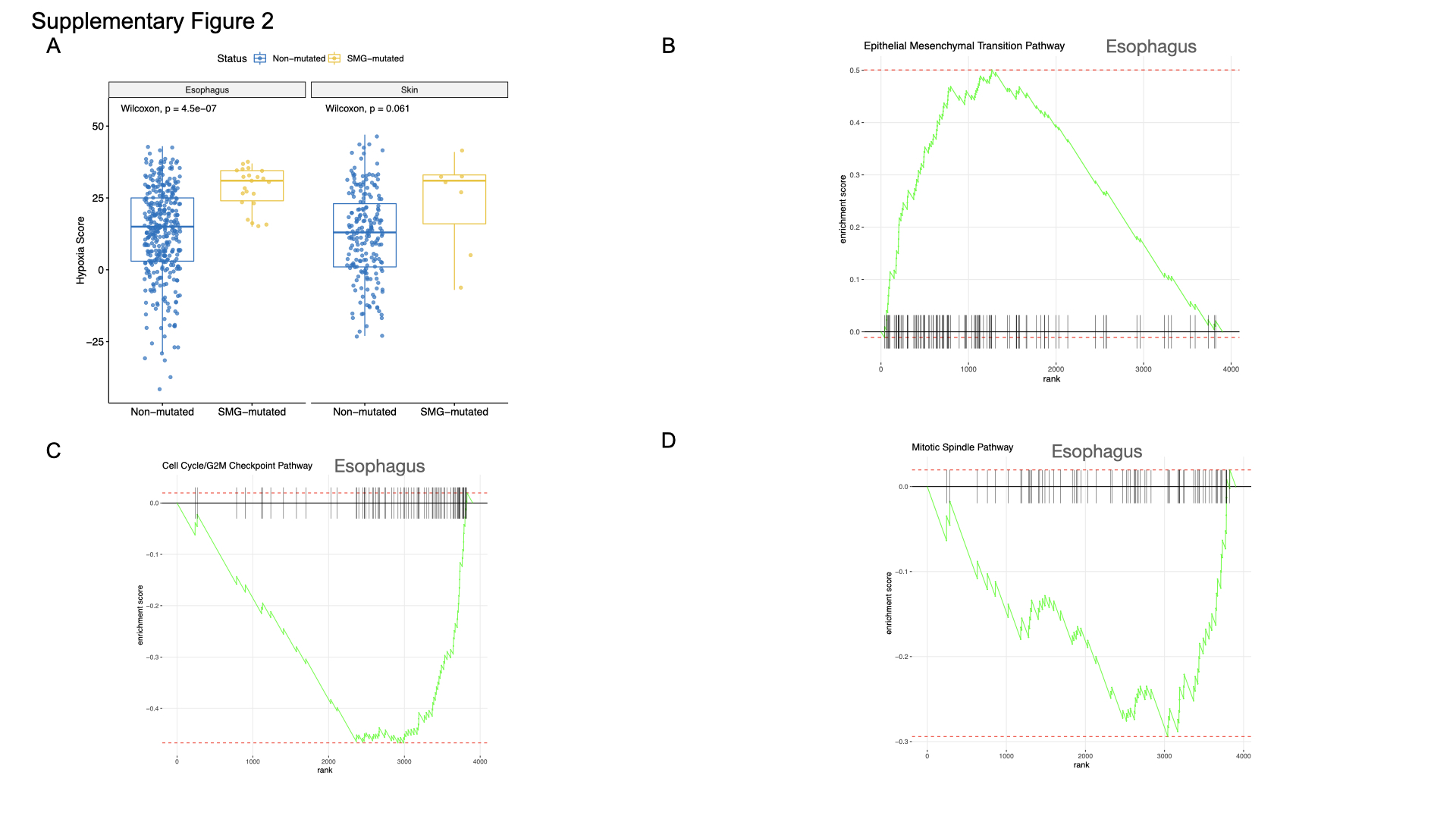

Supplement: Supplementary file 3 [file Image_2.jpeg]
